# (Dimeth­oxy­phosphor­yl)(furan-2-yl)methyl 2-(2,4-dichloro­phen­oxy)acetate

**DOI:** 10.1107/S1600536810042820

**Published:** 2010-10-30

**Authors:** Xiaosong Tan, Hao Peng, Hongwu He

**Affiliations:** a Key Laboratory of Pesticide and Chemical Biology, College of Chemistry, Central China Normal University, Wuhan 430079, People’s Republic of China

## Abstract

In the title compound, C_15_H_15_Cl_2_O_7_P, the benzene and furan rings form a dihedral angle of 73.54 (1)°. In the crystal, weak inter­molecular C—H⋯O hydrogen bonds link the mol­ecules into layers parallel to (100).

## Related literature

For the synthesis and biological activity of 1-(substituted phen­oxy­acet­oxy)alkyl­phospho­nate derivatives, see: He *et al.* (2001 ▶, 2005 ▶); Chen *et al.* (2006 ▶). The synthesis and biological activity of the title compound have been discussed by Peng *et al.* (2007 ▶).
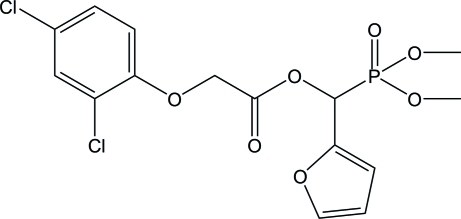


## Experimental

### 

#### Crystal data

C_15_H_15_Cl_2_O_7_P
*M* *_r_* = 409.14
Monoclinic, 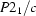

*a* = 8.5380 (7) Å
*b* = 17.3111 (14) Å
*c* = 12.4003 (10) Å
β = 98.475 (1)°
*V* = 1812.8 (3) Å^3^
*Z* = 4
Mo *K*α radiation
μ = 0.48 mm^−1^
*T* = 292 K
0.30 × 0.20 × 0.20 mm

#### Data collection

Bruker SMART APEX CCD area-detector diffractometer
12335 measured reflections
4135 independent reflections
3158 reflections with *I* > 2σ(*I*)
*R*_int_ = 0.048

#### Refinement

*R*[*F* ^2^ > 2σ(*F* ^2^)] = 0.049
*wR*(*F* ^2^) = 0.133
*S* = 1.05
4135 reflections
228 parameters
H-atom parameters constrained
Δρ_max_ = 0.36 e Å^−3^
Δρ_min_ = −0.31 e Å^−3^

### 

Data collection: *SMART* (Bruker, 2001 ▶); cell refinement: *SAINT* (Bruker, 2001 ▶); data reduction: *SAINT*; program(s) used to solve structure: *SHELXS97* (Sheldrick, 2008 ▶); program(s) used to refine structure: *SHELXL97* (Sheldrick, 2008 ▶); molecular graphics: *SHELXTL* (Sheldrick, 2008 ▶); software used to prepare material for publication: *SHELXTL*.

## Supplementary Material

Crystal structure: contains datablocks I, global. DOI: 10.1107/S1600536810042820/cv2778sup1.cif

Structure factors: contains datablocks I. DOI: 10.1107/S1600536810042820/cv2778Isup2.hkl

Additional supplementary materials:  crystallographic information; 3D view; checkCIF report

## Figures and Tables

**Table 1:** Hydrogen-bond geometry (Å, °)

*D*—H⋯*A*	*D*—H	H⋯*A*	*D*⋯*A*	*D*—H⋯*A*
C6—H6⋯O5^i^	0.93	2.50	3.322 (3)	148
C9—H9⋯O2^ii^	0.98	2.35	3.270 (2)	157
C14—H14*B*⋯O5^i^	0.96	2.44	3.379 (3)	165

Symmetry codes: (i) 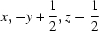; (ii) 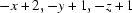.

**Fig. 1.**
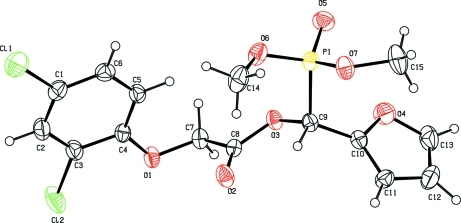
The molecular structure of the title compound, showing the atom-labeling scheme for the non-H atoms and 50% probability displacement ellipsoids.

**Fig. 2.**
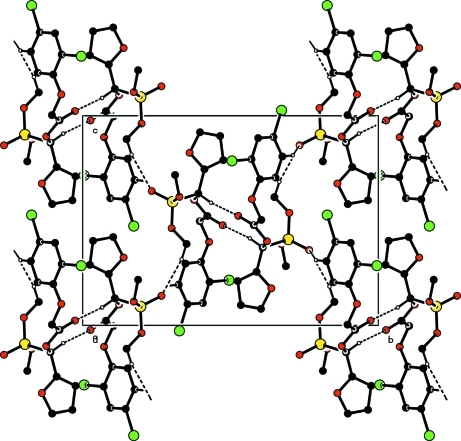
Part of the crystal packing, showing the C—H···O hydrogen bonds as dashed lines.

## supplementary crystallographic information

### Comment

Phosphonate derivatives are particularly important in connection with their remarkable biological activities. They have been widely used as enzyme inhibitors, antibacterial agents, anti-HIV agents, and also used as pesticides. As a continuation of our search for novel herbicides, series of 1-(substituted phenoxyacetoxy)alkylphosphonate derivatives have been designed and synthesized. Some of them have shown good herbicidal activities acting as inhibitors of PDHc (He *et al.*, 2001, 2005; Chen *et al.*, 2006; Peng *et al.*, 2007). The title compound (I) can be used as herbicide to control broadleaf weeds and sedge weeds effectively and showed good selectivity between monocotyledonous crops and dicotyledonous weeds. Here, we report the crystal structure of (I).

The title compound (Fig. 1), has formed a racemate crystal with monoclinic (*P*2_1_/*c*) symmetry. The benzene and furan rings in the molecule are nonplanar, and the dihedral angle between the two rings is 73.54 (1)°.

In the crystal structure, weak intermolecular C—H···O hydrogen bonds (Table 1) link the molecules into layers parallel to the (100) plane. Weak C—H···π interaction (C15···*Cg*1 = 3.690 (3) Å, C15—H15···*Cg*1=137°, symmetry code: *x*, *y*, *z*, *Cg*1 is the centroid defined by atoms O4/C10—C13) was also observed in the crystal structure.

### Experimental

A solution of 2,4-dichorophenoxyacetyl chloride (11 mmol) in trichloromethane (15 ml) was added to stirred mixture of 1-hydroxy (furan-2-yl)methylphosphonate (10 mmol) and triethyl amine (11 mmol) in trichloromethane (15 ml) at 273 K. The resultant mixture was stirred at ambient temperature for 3 h, then washed with dilute hydrochloric acid solution, saturated sodium hydrogen carbonate and brine separately, dried and evaporated. The residue was chromatographed on silica with acetone and petroleum ether as eluent to give the title compound as a white solid.

### Refinement

All hydrogen atoms were geometrically positioned [C—H 0.93-0.98 Å],  and refined as riding, with *U*_iso_(H) = 1.2-1.5 *U*_eq_(C).

### Crystal data

**Table:**

C_15_H_15_Cl_2_O_7_P	*F*(000) = 840
*M**_r_* = 409.14	*D*_x_ = 1.499 Mg m^−^^3^
Monoclinic, *P*2_1_/*c*	Mo *K*α radiation, λ = 0.71073 Å
Hall symbol: -P 2ybc	Cell parameters from 4066 reflections
*a* = 8.5380 (7) Å	θ = 2.4–28.2°
*b* = 17.3111 (14) Å	µ = 0.48 mm^−^^1^
*c* = 12.4003 (10) Å	*T* = 292 K
β = 98.475 (1)°	Block, colourless
*V* = 1812.8 (3) Å^3^	0.30 × 0.20 × 0.20 mm
*Z* = 4	

### Data collection

**Table:**

Bruker SMART APEX CCD area-detector diffractometer	3158 reflections with *I* > 2σ(*I*)
Radiation source: fine-focus sealed tube	*R*_int_ = 0.048
graphite	θ_max_ = 27.5°, θ_min_ = 2.0°
phi and ω scans	*h* = −11→11
12335 measured reflections	*k* = −22→19
4135 independent reflections	*l* = −15→15

### Refinement

**Table:**

Refinement on *F*^2^	Primary atom site location: structure-invariant direct methods
Least-squares matrix: full	Secondary atom site location: difference Fourier map
*R*[*F*^2^ > 2σ(*F*^2^)] = 0.049	Hydrogen site location: inferred from neighbouring sites
*wR*(*F*^2^) = 0.133	H-atom parameters constrained
*S* = 1.05	*w* = 1/[σ^2^(*F*_o_^2^) + (0.0672*P*)^2^ + 0.2806*P*] where *P* = (*F*_o_^2^ + 2*F*_c_^2^)/3
4135 reflections	(Δ/σ)_max_ = 0.001
228 parameters	Δρ_max_ = 0.36 e Å^−^^3^
0 restraints	Δρ_min_ = −0.31 e Å^−^^3^

### Special details

**Table:**

Geometry. All e.s.d.'s (except the e.s.d. in the dihedral angle between two l.s. planes) are estimated using the full covariance matrix. The cell e.s.d.'s are taken into account individually in the estimation of e.s.d.'s in distances, angles and torsion angles; correlations between e.s.d.'s in cell parameters are only used when they are defined by crystal symmetry. An approximate (isotropic) treatment of cell e.s.d.'s is used for estimating e.s.d.'s involving l.s. planes.
Refinement. Refinement of *F*^2^ against ALL reflections. The weighted *R*-factor *wR* and goodness of fit *S* are based on *F*^2^, conventional *R*-factors *R* are based on *F*, with *F* set to zero for negative *F*^2^. The threshold expression of *F*^2^ > σ(*F*^2^) is used only for calculating *R*-factors(gt) *etc*. and is not relevant to the choice of reflections for refinement. *R*-factors based on *F*^2^ are statistically about twice as large as those based on *F*, and *R*- factors based on ALL data will be even larger.

### Fractional atomic coordinates and isotropic or equivalent isotropic displacement parameters (Å^2^)

**Table:**

	*x*	*y*	*z*	*U*_iso_*/*U*_eq_	
C1	0.6128 (3)	0.35463 (14)	0.10119 (19)	0.0489 (5)	
C2	0.4929 (3)	0.40648 (14)	0.10635 (19)	0.0545 (6)	
H2	0.4350	0.4262	0.0429	0.065*	
C3	0.4592 (3)	0.42900 (14)	0.20734 (19)	0.0474 (5)	
C4	0.5448 (2)	0.40033 (12)	0.30291 (17)	0.0377 (4)	
C5	0.6644 (2)	0.34767 (12)	0.29467 (18)	0.0411 (5)	
H5	0.7220	0.3274	0.3578	0.049*	
C6	0.7000 (3)	0.32463 (13)	0.1940 (2)	0.0459 (5)	
H6	0.7812	0.2896	0.1892	0.055*	
C7	0.5786 (2)	0.39588 (13)	0.49683 (17)	0.0412 (5)	
H7A	0.5747	0.3400	0.4922	0.049*	
H7B	0.5227	0.4116	0.5559	0.049*	
C8	0.7491 (2)	0.42157 (12)	0.52171 (16)	0.0361 (4)	
C9	0.9975 (2)	0.39109 (12)	0.62871 (16)	0.0361 (4)	
H9	1.0269	0.4344	0.5849	0.043*	
C10	1.0363 (2)	0.41187 (12)	0.74493 (17)	0.0390 (5)	
C11	1.1050 (3)	0.47563 (13)	0.78872 (19)	0.0449 (5)	
H11	1.1352	0.5186	0.7517	0.054*	
C12	1.1229 (3)	0.46505 (18)	0.9034 (2)	0.0631 (7)	
H12	1.1684	0.4998	0.9560	0.076*	
C13	1.0633 (3)	0.39691 (18)	0.9214 (2)	0.0642 (7)	
H13	1.0593	0.3757	0.9898	0.077*	
C14	1.1220 (4)	0.35140 (18)	0.3883 (2)	0.0681 (8)	
H14A	1.0862	0.4032	0.3976	0.102*	
H14B	1.0867	0.3348	0.3149	0.102*	
H14C	1.2355	0.3499	0.4026	0.102*	
C15	1.3707 (3)	0.3126 (2)	0.7176 (2)	0.0762 (9)	
H15A	1.3491	0.3517	0.7684	0.114*	
H15B	1.4810	0.3138	0.7104	0.114*	
H15C	1.3442	0.2628	0.7438	0.114*	
Cl1	0.65651 (11)	0.32723 (5)	−0.02623 (6)	0.0777 (3)	
Cl2	0.30927 (9)	0.49516 (5)	0.21544 (6)	0.0819 (3)	
O1	0.50190 (16)	0.42765 (9)	0.39781 (12)	0.0444 (4)	
O2	0.80530 (19)	0.47377 (9)	0.47743 (14)	0.0548 (4)	
O3	0.82710 (15)	0.37669 (8)	0.59892 (11)	0.0394 (3)	
O4	1.0077 (2)	0.36170 (10)	0.82338 (14)	0.0610 (5)	
O5	1.0513 (2)	0.23366 (9)	0.64065 (14)	0.0552 (4)	
O6	1.0582 (2)	0.30095 (10)	0.46306 (13)	0.0546 (4)	
O7	1.27695 (17)	0.32711 (10)	0.61288 (14)	0.0510 (4)	
P1	1.09650 (6)	0.30439 (3)	0.58986 (5)	0.04063 (17)	

### Atomic displacement parameters (Å^2^)

**Table:**

	*U*^11^	*U*^22^	*U*^33^	*U*^12^	*U*^13^	*U*^23^
C1	0.0557 (13)	0.0517 (14)	0.0399 (12)	−0.0049 (11)	0.0090 (10)	−0.0065 (10)
C2	0.0591 (14)	0.0644 (16)	0.0368 (13)	0.0079 (12)	−0.0036 (11)	0.0024 (11)
C3	0.0411 (11)	0.0545 (13)	0.0442 (13)	0.0110 (10)	−0.0014 (10)	0.0008 (11)
C4	0.0301 (9)	0.0439 (11)	0.0377 (11)	−0.0011 (8)	0.0003 (8)	−0.0009 (9)
C5	0.0352 (10)	0.0446 (12)	0.0415 (12)	0.0010 (9)	−0.0015 (9)	0.0027 (9)
C6	0.0425 (11)	0.0432 (12)	0.0522 (14)	0.0026 (9)	0.0082 (10)	−0.0050 (10)
C7	0.0357 (10)	0.0517 (13)	0.0356 (11)	−0.0018 (9)	0.0031 (8)	0.0030 (9)
C8	0.0373 (10)	0.0402 (11)	0.0301 (10)	−0.0008 (8)	0.0023 (8)	−0.0016 (8)
C9	0.0308 (9)	0.0402 (11)	0.0362 (11)	−0.0047 (8)	0.0018 (8)	0.0062 (9)
C10	0.0356 (10)	0.0443 (12)	0.0369 (11)	0.0040 (9)	0.0046 (9)	0.0037 (9)
C11	0.0509 (13)	0.0404 (12)	0.0436 (13)	−0.0089 (10)	0.0070 (10)	−0.0034 (10)
C12	0.0587 (15)	0.0773 (19)	0.0502 (15)	0.0000 (14)	−0.0022 (12)	−0.0206 (14)
C13	0.0732 (17)	0.084 (2)	0.0352 (13)	0.0139 (16)	0.0064 (12)	0.0061 (13)
C14	0.0799 (19)	0.088 (2)	0.0378 (14)	−0.0214 (16)	0.0133 (13)	0.0000 (13)
C15	0.0442 (14)	0.116 (3)	0.0642 (19)	0.0139 (15)	−0.0057 (13)	0.0160 (17)
Cl1	0.1028 (6)	0.0855 (5)	0.0477 (4)	0.0091 (4)	0.0213 (4)	−0.0122 (3)
Cl2	0.0755 (5)	0.1100 (6)	0.0563 (4)	0.0552 (4)	−0.0029 (3)	0.0040 (4)
O1	0.0355 (7)	0.0607 (10)	0.0353 (8)	0.0096 (7)	−0.0004 (6)	−0.0011 (7)
O2	0.0500 (9)	0.0558 (10)	0.0541 (10)	−0.0142 (8)	−0.0072 (7)	0.0202 (8)
O3	0.0322 (7)	0.0458 (8)	0.0389 (8)	−0.0049 (6)	0.0004 (6)	0.0105 (6)
O4	0.0774 (12)	0.0568 (11)	0.0505 (10)	−0.0025 (9)	0.0147 (9)	0.0053 (8)
O5	0.0657 (10)	0.0453 (9)	0.0558 (10)	−0.0021 (8)	0.0123 (8)	0.0066 (8)
O6	0.0633 (10)	0.0619 (10)	0.0387 (9)	−0.0138 (8)	0.0074 (8)	−0.0046 (7)
O7	0.0370 (8)	0.0686 (11)	0.0468 (9)	0.0026 (7)	0.0043 (7)	0.0063 (8)
P1	0.0402 (3)	0.0448 (3)	0.0369 (3)	−0.0021 (2)	0.0060 (2)	0.0002 (2)

### Geometric parameters (Å, °)

**Table:**

C1—C2	1.370 (3)	C9—H9	0.9800
C1—C6	1.377 (3)	C10—C11	1.328 (3)
C1—Cl1	1.742 (2)	C10—O4	1.353 (2)
C2—C3	1.382 (3)	C11—C12	1.419 (3)
C2—H2	0.9300	C11—H11	0.9300
C3—C4	1.389 (3)	C12—C13	1.317 (4)
C3—Cl2	1.732 (2)	C12—H12	0.9300
C4—O1	1.367 (2)	C13—O4	1.380 (3)
C4—C5	1.384 (3)	C13—H13	0.9300
C5—C6	1.386 (3)	C14—O6	1.438 (3)
C5—H5	0.9300	C14—H14A	0.9600
C6—H6	0.9300	C14—H14B	0.9600
C7—O1	1.415 (2)	C14—H14C	0.9600
C7—C8	1.509 (3)	C15—O7	1.444 (3)
C7—H7A	0.9700	C15—H15A	0.9600
C7—H7B	0.9700	C15—H15B	0.9600
C8—O2	1.194 (2)	C15—H15C	0.9600
C8—O3	1.333 (2)	O5—P1	1.4548 (16)
C9—O3	1.468 (2)	O6—P1	1.5591 (17)
C9—C10	1.475 (3)	O7—P1	1.5749 (16)
C9—P1	1.822 (2)		
			
C2—C1—C6	121.6 (2)	C11—C10—C9	128.6 (2)
C2—C1—Cl1	118.84 (19)	O4—C10—C9	120.50 (19)
C6—C1—Cl1	119.56 (19)	C10—C11—C12	106.2 (2)
C1—C2—C3	118.9 (2)	C10—C11—H11	126.9
C1—C2—H2	120.5	C12—C11—H11	126.9
C3—C2—H2	120.5	C13—C12—C11	107.3 (2)
C2—C3—C4	121.3 (2)	C13—C12—H12	126.3
C2—C3—Cl2	119.57 (18)	C11—C12—H12	126.3
C4—C3—Cl2	119.15 (18)	C12—C13—O4	109.7 (2)
O1—C4—C5	125.83 (19)	C12—C13—H13	125.1
O1—C4—C3	115.91 (19)	O4—C13—H13	125.1
C5—C4—C3	118.3 (2)	O6—C14—H14A	109.5
C4—C5—C6	121.2 (2)	O6—C14—H14B	109.5
C4—C5—H5	119.4	H14A—C14—H14B	109.5
C6—C5—H5	119.4	O6—C14—H14C	109.5
C1—C6—C5	118.7 (2)	H14A—C14—H14C	109.5
C1—C6—H6	120.6	H14B—C14—H14C	109.5
C5—C6—H6	120.6	O7—C15—H15A	109.5
O1—C7—C8	111.83 (16)	O7—C15—H15B	109.5
O1—C7—H7A	109.2	H15A—C15—H15B	109.5
C8—C7—H7A	109.2	O7—C15—H15C	109.5
O1—C7—H7B	109.2	H15A—C15—H15C	109.5
C8—C7—H7B	109.2	H15B—C15—H15C	109.5
H7A—C7—H7B	107.9	C4—O1—C7	117.64 (16)
O2—C8—O3	125.38 (18)	C8—O3—C9	116.97 (15)
O2—C8—C7	124.74 (19)	C10—O4—C13	105.9 (2)
O3—C8—C7	109.87 (17)	C14—O6—P1	125.73 (16)
O3—C9—C10	111.03 (15)	C15—O7—P1	121.16 (16)
O3—C9—P1	105.95 (13)	O5—P1—O6	112.02 (9)
C10—C9—P1	114.39 (14)	O5—P1—O7	116.52 (10)
O3—C9—H9	108.4	O6—P1—O7	104.26 (9)
C10—C9—H9	108.4	O5—P1—C9	114.57 (9)
P1—C9—H9	108.4	O6—P1—C9	105.17 (9)
C11—C10—O4	110.8 (2)	O7—P1—C9	103.06 (9)
			
C6—C1—C2—C3	0.0 (4)	C5—C4—O1—C7	−4.5 (3)
Cl1—C1—C2—C3	−179.22 (19)	C3—C4—O1—C7	175.69 (18)
C1—C2—C3—C4	0.2 (4)	C8—C7—O1—C4	71.0 (2)
C1—C2—C3—Cl2	179.42 (19)	O2—C8—O3—C9	−2.4 (3)
C2—C3—C4—O1	179.2 (2)	C7—C8—O3—C9	176.68 (16)
Cl2—C3—C4—O1	0.0 (3)	C10—C9—O3—C8	119.82 (19)
C2—C3—C4—C5	−0.6 (3)	P1—C9—O3—C8	−115.43 (16)
Cl2—C3—C4—C5	−179.82 (16)	C11—C10—O4—C13	−0.4 (3)
O1—C4—C5—C6	−178.97 (19)	C9—C10—O4—C13	177.36 (19)
C3—C4—C5—C6	0.8 (3)	C12—C13—O4—C10	0.0 (3)
C2—C1—C6—C5	0.2 (4)	C14—O6—P1—O5	−163.4 (2)
Cl1—C1—C6—C5	179.39 (16)	C14—O6—P1—O7	−36.6 (2)
C4—C5—C6—C1	−0.6 (3)	C14—O6—P1—C9	71.5 (2)
O1—C7—C8—O2	14.4 (3)	C15—O7—P1—O5	−36.0 (2)
O1—C7—C8—O3	−164.71 (17)	C15—O7—P1—O6	−160.0 (2)
O3—C9—C10—C11	−121.5 (2)	C15—O7—P1—C9	90.4 (2)
P1—C9—C10—C11	118.6 (2)	O3—C9—P1—O5	−57.93 (15)
O3—C9—C10—O4	61.2 (2)	C10—C9—P1—O5	64.71 (17)
P1—C9—C10—O4	−58.7 (2)	O3—C9—P1—O6	65.52 (14)
O4—C10—C11—C12	0.6 (3)	C10—C9—P1—O6	−171.84 (14)
C9—C10—C11—C12	−176.9 (2)	O3—C9—P1—O7	174.48 (12)
C10—C11—C12—C13	−0.6 (3)	C10—C9—P1—O7	−62.88 (16)
C11—C12—C13—O4	0.4 (3)		

### Hydrogen-bond geometry (Å, °)

**Table:**

*D*—H···*A*	*D*—H	H···*A*	*D*···*A*	*D*—H···*A*
C6—H6···O5^i^	0.93	2.50	3.322 (3)	148
C9—H9···O2^ii^	0.98	2.35	3.270 (2)	157
C14—H14B···O5^i^	0.96	2.44	3.379 (3)	165

Symmetry codes: (i) *x*, −*y*+1/2, *z*−1/2; (ii) −*x*+2, −*y*+1, −*z*+1.
